# Novel Antimicrobial Organic Thermal Stabilizer and Co-Stabilizer for Rigid PVC

**DOI:** 10.3390/molecules17077927

**Published:** 2012-07-02

**Authors:** Mona M. Fahmy, Riham R. Mohamed, Nadia A. Mohamed

**Affiliations:** Department of Chemistry, Faculty of Science, Cairo University, Giza 12613, Egypt

**Keywords:** PVC, thermal stability, discoloration, antimicrobial activity

## Abstract

Biologically active *N*-benzoyl-4-(*N*-maleimido)-phenylhydrazide (BMPH) was synthesized and its structure was confirmed by elemental analysis and various spectral tools. It was examined as a thermal stabilizer and co-stabilizer for rigid poly (vinyl chloride) at 180 °C in air. Blending BMPH with reference samples in different ratios greatly lengthens the thermal stability value and improves the extent of discoloration of PVC. TGA confirmed the improved stability of PVC in presence of the investigated organic stabilizer. GPC measurements were done to investigate the changes occurred in the molecular masses of the degraded samples of blank PVC and PVC in presence of the novel stabilizer. BMPH showed good antimicrobial activity towards two kinds of bacteria and two kinds of fungi.

## 1. Introduction

It is generally known that poly (vinyl chloride), PVC, is an unstable polymer when exposed to high temperatures during its molding and applications. PVC undergoes extensive autocatalytic dehydrochlorination with formation of conjugated double bonds [1]. This in turn leads to unacceptable discoloration of the polymer and changes in its physical and mechanical properties [2,3,4], together with a decrease or an increase in its average molecular weight as a result of chain scission or crosslinking. It has been generally considered that thermal dehydrochlorination is initiated by structural defects such as allylic chlorine associated with internal unsaturation [5], tertiary hydrogen and chlorine atoms associated with branches [6], reactive terminal groups such as double bonds [5], oxygen-containing groups [7], or peroxide residues [8] and head-to-head structures [9].

Thus, stabilization of PVC against thermal degradation is essential for its processing and use at high temperatures. The thermal stabilizers commonly in use for the stabilization of PVC are either basic lead salts [10], which can react with the evolved HCl gas, thus retarding its deleterious catalytic action, metallic soaps [11] and esters or mercaptides of dialkyltin [12] that can exchange the labile chlorine in the backbone chains for other more stable ester or mercaptide groups derived from the stabilizer. 

In spite of the fact that the aforementioned classes of stabilizers are quite efficient industrially, the metallic residues of some of these organometallic compounds (lead, cadmium or tin) may present serious environmental problems. Others, such as zinc, form chloride salts which are strong catalysts for the subsequent dehydrochlorination process, and are responsible for the sudden blackening of certain formulations. For this reason, the attention of many investigators has been directed recently to stabilizers of a fully organic nature [13,14,15,16,17,18,19,20,21,22,23,24,25,26,27]. Moreover, studies were performed to prepare antibacterial PVC. These trials were based on surface modification of PVC using different nanoparticles to prepare PVC/antibacterial composites. Zirconium phosphate [28] and TiO_2_/Ag [29] were used for this purpose. Isothiocyanate nucleophilically substituted PVC was also used to obtain an antibacterial PVC [30]. On the other hand, pyrazolodithiones with antimicrobial and antitumor activity have been used recently as additives for thermal stabilization of rigid PVC [26].

*N*-substituted maleimides [31] and aromatic hydrazides [32] have been proven to be effective additives for PVC stabilization against thermal degradation. However, the aromatic hydrazides act as HCl absorbers; the maliemides act as powerful radical traps and can exchange the labile chlorine in the PVC chains for a more stable maleimide moiety.

Thus, it became of interest to study the thermal stability of PVC in the presence of a novel organic stabilizer, *N*-benzoyl-4-(*N*-maleimido)-phenylhydrazide (BMPH), which combines the characteristics of both the maleimide and hydrazide groups in its structure. BMPH was prepared for the first time in this work and its structure was confirmed by elemental analysis and several spectral tools. It was also characterized for its antimicrobial activity.

## 2. Results and Discussion

### 2.1. Preparation and Characterization of N-benzoyl-4-(N-maleimido)-phenylhydrazide (BMPH)

**Scheme 1:**
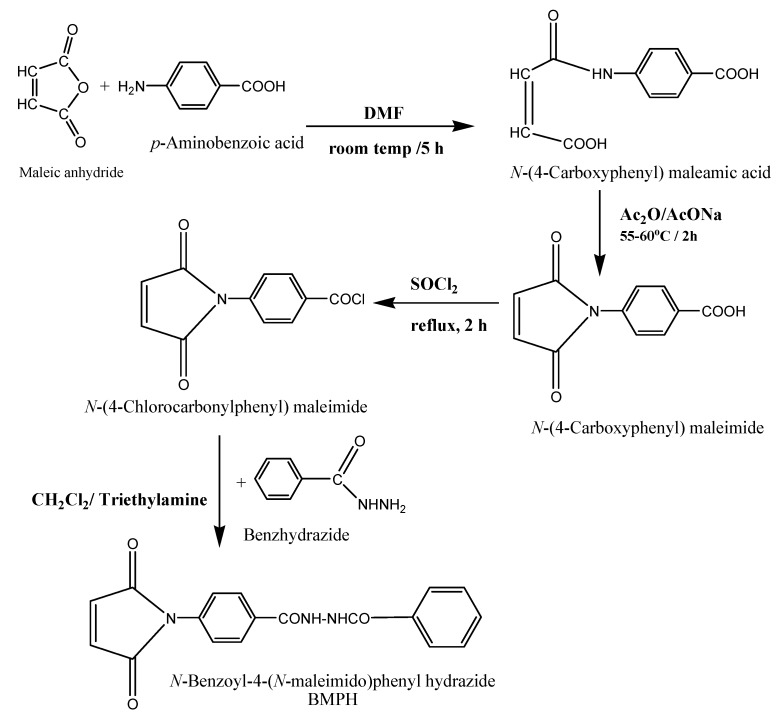
The synthesis of BMPH.

BMPH was synthesized from maleic anhydride, *p*-aminobenzoic acid and benzhydrazide, as shown in Scheme 1.

BMPH: Yield: 75.8%; m.p. = 164–166 °C. Elemental analyses: *Theor*. C (64.47%), H (3.91%), N (12.53%) and O (19.09%); *Exp*. C (64.04%), H (3.86%), N (12.13%) and O (18.98%).

**Figure 1:**
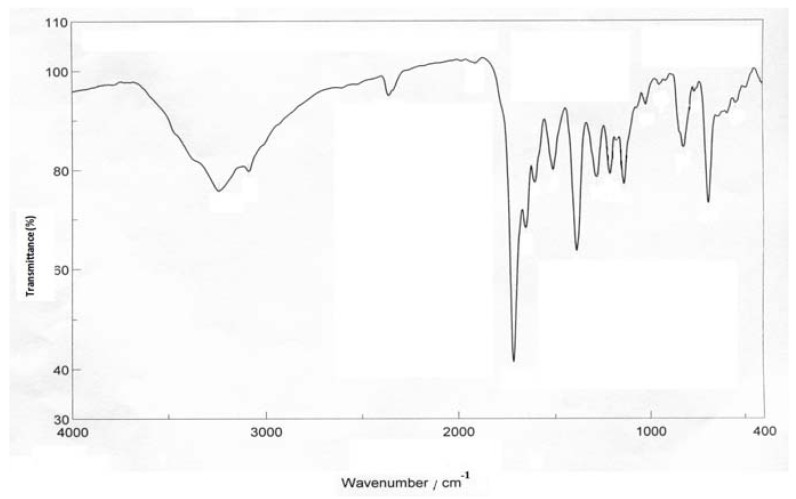
FTIR spectrum of BMPH.

FTIR spectrum of BMPH (Figure 1) showed a specific band for maleimide moiety at 830 cm^−1^, while two strong bands appeared at 1509 and 1603 cm^−1^ corresponding to the stretching vibration for aromatic rings. Moreover, a strong band for the stretching vibration of carbonyl groups of hydrazide linkage appeared at 1650 cm^−1^. A specific band appeared at 1714 cm^−1^ for the C=O of the imide linkages of the maleimide moiety. Also the -NH- stretching band appeared at 3239 cm^−1^.

**Figure 2:**
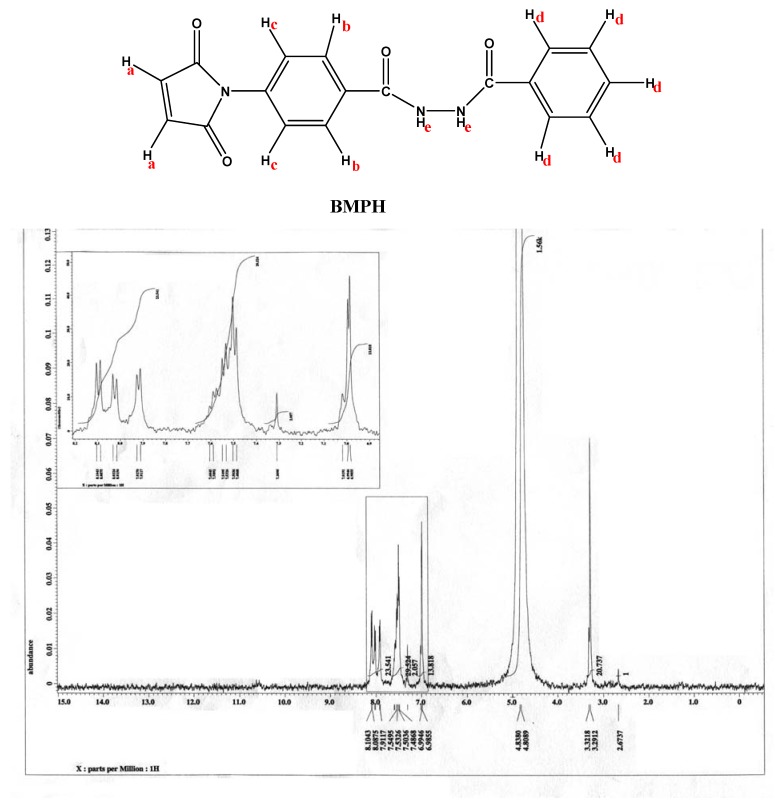
^1^H-NMR spectrum of BMPH.

^1^H-NMR spectrum of BMPH (Figure 2) (DMSO): δ_He_ = 7.0 ppm. (2H, -NH-); δ_Hd_ = 7.5–7.6 ppm (5H, aromatic protons); δ_Hc_ = 7.9 ppm (2H, *o*-H); δ_Hb_ = 8.0 ppm (2H, *m*-H), δ_Ha_ = 8.1 ppm (2H, H-C=C-H). MS *m/z*: 335 (M^+^).

### 2.2. Stabilization of Thermally Degraded Rigid PVC Using BMPH

**Figure 3:**
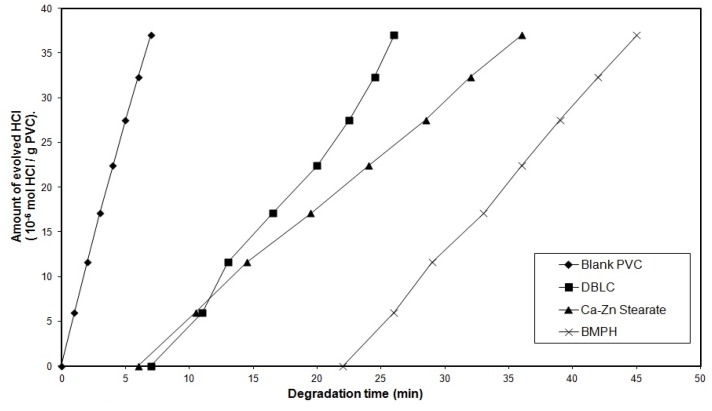
Dehydrochlorination rate of rigid PVC (DBLC, Ca-Zn stearate, BMPH) at 180 °C, in air, in the presence of 2 mass % of BMPH stabilizer and reference stabilizers.

Results of the dehydrochlorination of rigid PVC thermally degraded at 180 °C in air, in the presence of the investigated stabilizer are shown both in Figure 3 and Table 1. The results of the non-stabilized blank PVC as well as those of the samples stabilized by DBLC and Ca–Zn stearate used as reference stabilizers are also given for comparison. The results reveal that BMPH exhibits a greater stabilizing efficiency than those of the two reference stabilizers, which is shown by the longer thermal stability value (Ts) *during which no detectable amounts of hydrogen chloride gas are liberated* (Table 1). The thermal stability value of BMPH is almost three or four times higher than the values obtained for the reference stabilizers.

It has been previously suggested that maleimide derivatives owe their stabilizing efficiency to the replacement of the labile chlorine atoms on PVC chains by a relatively more thermally stable stabilizer moiety. The stabilizers’ efficiency is attributed to their radical potency, which interferes with the PVC radical degradation process. This most probably occurs not only through trapping the radical species in the degradation process, but also by blocking the radical sites created on PVC chains. The radical attack seems to occur first on the ethylenic carbon–carbon double bond, followed by cleavage of the imide linkages during the later stages of degradation. This mode of action has previously been published, together with the experiments to prove it [31]. Furthermore, aromatic hydrazides are efficient thermal stabilizers for rigid PVC. They exhibit their stabilizing efficiency through effective absorption of the degradation products (HCl gas) by their basic groups [32].

In view of structural similarity of BMPH to maleimides combined with aromatic hydrazides, the mechanism of BMPH is outlined by assuming that its first part (maleimide) can work as a radical trapper, while its other part (benzhydrazide) acts as HCl absorber.

Other experimental evidence for the high stabilizing efficiency of BMPH is illustrated by the improvement in the extent of discoloration of PVC samples stabilized with BMPH and thermally degraded at 180 °C in air for different time intervals relative to blank PVC sample and PVC samples stabilized with any of the reference stabilizers (Table 2). This reflects the greater stabilizing efficiency of BMPH through the replacement of the labile chlorine by a more thermally stable stabilizer moiety which disturbs the formation of the conjugated double bonds that are responsible for discoloration.

Moreover, the improvement in the extent of discoloration of PVC in presence of BMPH is most probably due to its dienophilic properties, which allow it to intervene with the conjugated double bond systems formed by Diels-Alder addition on PVC chains during the subsequent stages of the degradation process. Several investigators have attributed the good color stability of the dibutyltin maleate stabilizer to the same type of addition reaction [33].

### 2.3. Elucidation of the Molecular Mass by GPC

GPC measurements were carried out for both PVC, before and after 30 min of thermal degradation in presence or absence of BMPH stabilizer. Results are summarized in Table 3, where the values of M_w_, M_n_ and polydisperity (PD) are shown.

The GPC measurement results indicate the low decrease in the values of molecular masses of PVC samples achieved by using BMPH as thermal stabilizer. The results of GPC measurements show the decrease in M_w_ value of blank PVC sample from 2.4473 × 10^5^ to 1.8702 × 10^5^ upon 30 min of thermal degradation with a % decrease in M_w_ value of 23.58. After the same time of degradation, the decrease in M_w_ of a PVC sample stabilized with BMPH reaches only 15.94%.

This may be due to the good stabilizing effect of the investigated compound that decreases the extent of chain scission of PVC. The solubility test of thermally degraded PVC indicates the absence of gel formation, which indicates the absence of cross linking during degradation. This is evidence for the high efficiency for the investigated stabilizer, that it can decrease the chain scission and prevent cross linking, so it can preserve both the mechanical and physical properties of the polymer.

### 2.4. Thermogravimetric Analysis

Thermal stability and degradation behavior for blank PVC and PVC stabilized with BMPH were investigated by TG measurements. The results obtained are given in Table 4. BMPH greatly improved the initial decomposition temperature of PVC, as the IDT of PVC stabilized with BMPH was recorded as 265 °C, whereas the IDT of the non-stabilized PVC was found to be 180 °C. Furthermore, at all temperatures used, PVC samples stabilized with BMPH showed higher stability (lower mass loss %) relative to that of blank PVC. These results suggest that the degradation rate of PVC sample stabilized with BMPH is slower than that of blank PVC sample. From the aforementioned results, it was clear that BMPH stabilizer had increased the thermal stability of PVC.

### 2.5. Effect of Mixed Stabilizers on the Stabilization Efficiency of Thermally Degraded Rigid PVC

**Figure 4:**
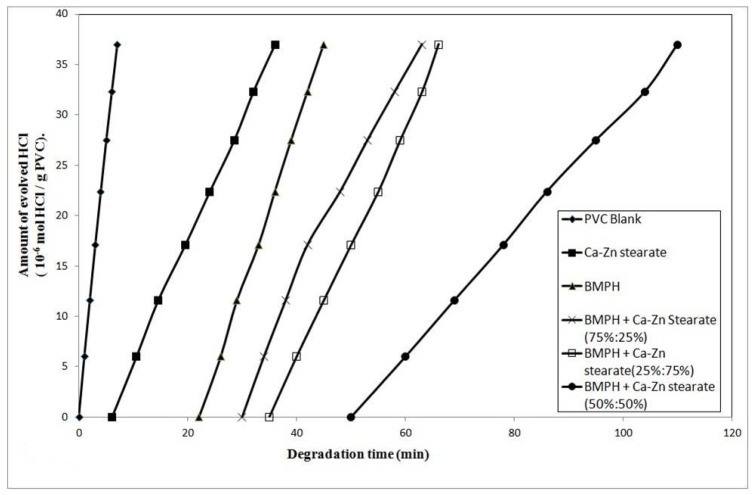
Dehydrochlorination rate of rigid PVC (BMPH stabilizer was mixed with Ca-Zn stearate), at 180 °C, in air, in the presence of 2 mass % of mixed stabilizers.

**Figure 5:**
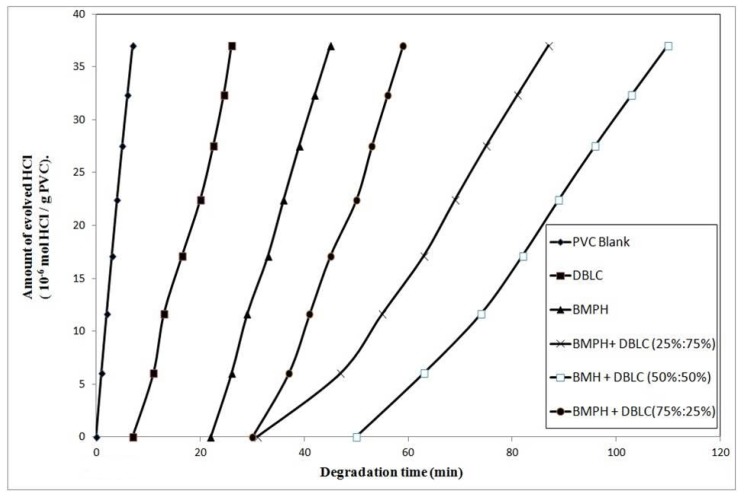
Dehydrochlorination rate of rigid PVC (BMPH stabilizer was mixed with DBLC), at 180 °C, in air, in the presence of 2 mass % of mixed stabilizers.

It was of interest to study the effect on the efficiency of stabilization of mixing BMPH stabilizer with reference stabilizers. Mixing was done in the ranges of 0–100% of BMPH relative to DBLC or Ca-Zn stearate reference stabilizers. The overall mixed stabilizers concentration was kept always constant at 2 mass % of PVC and the results represent the average of three comparable experiments for each stabilizer mixture. The results of the dehydrochlorination of thermally degraded rigid PVC at 180 °C, in air, in the presence of each combination are illustrated in Figure 4 and Figure 5. The thermal stability values of such combinations are listed in Table 1. The results reveal the existence of a true synergistic effect resulting from the combination of BMPH stabilizer with any of the two reference stabilizers.

The maximum synergism was achieved when the BMPH stabilizer was mixed with DBLC or Ca–Zn stearate in an equivalent weight ratio. The results also demonstrated a slight improvement in the rate of dehydrochlorination as a result of mixing the investigated stabilizer with the reference stabilizers.

It has been established that the hydrazide linkage can interact with various transition metal salts to form stable complexes [27]. For this, at subsequent stages of degradation, it would be possible for the hydrazide moiety of BMPH stabilizer to react with the accumulated metal chlorides (ZnCl_2_ or PbCl_2_) formed as by-products from reference stabilizers to form complexes with additional stabilizing power and improve the stabilizing efficiency of the mixed stabilizers. Thus, mixing the reference stabilizers with BMPH, would not only remove the deleterious effect of the metal chlorides but also it give an additional advantage from their transformation into useful new stabilizers.

An additional proof for the synergistic effect achieved by mixing the investigated stabilizer with the reference stabilizers on the extent of discoloration of rigid PVC thermally degraded at 180 °C, in air, for 60 min (Table 5). The results clearly reveal that all the mixed stabilizers exhibit a reduction of the discoloration of the PVC than the reference stabilizers, rather than the BMPH, when they are used separately. The least extent of discoloration was achieved when the BMPH and reference stabilizers were mixed in equivalent weight ratio.

### 2.6. Biological Activity for BMPH against Bacterial and Fungal Strains Isolated from Animal Origin

BMPH showed antibacterial activity of 52% of that of the reference antibacterial agent (ciprofloxacin) against *S. pneumonia*, about 40% of ciprofloxacin against *S. aureus*, 60% of ciprofloxacin against *S. typhimurium* and 58.8% of ciprofloxacin against *E. coli* bacteria (Table 6). Also, it has antifungal activity as shown in Table 6. This activity represents 27% and 46.7% of the reference antifungal agent (flucanazole) against two kinds of fungi; *A. flavus* and *C. albicans*, respectively.

Thus, BMPH stabilizer exhibited reasonable biological activity due to the presence of either -NH or -C=O groups in its structure and it is clear that its antibacterial activity is higher than its antifungal activity.

## 3. Experimental

### 3.1. General

The commercial PVC (suspension) used in this study was additive free, with a K value of 70 and supplied by Hüls Co. (Frankfurt, Germany). Calcium–zinc stearate complex (Ca-Zn stearate) obtained from G. Siegle Co. (Stuttgart, Germany), and dibasic lead carbonate (DBLC) (Rolite lead) obtained from the National Lead Co. (Darmstadt, Germany), were also used. The FTIR spectrum was recorded on a Shimadzu FT-IR 8201 PC Spectrophotometer using KBr pellets. The ^1^H-NMR spectrum was recorded with a Jeol 270 MHz (Tokyo, Japan) spectrometer in DMSO-d_6_ as a solvent and the chemical shifts were recorded in ppm relative to TMS as an internal standard. Mass spectra were recorded on GCMS-QP 1000 ex spectra mass spectrometer (Shimadzu, Tokyo, Japan) operating at 70 eV. Elemental analyses were carried out by the micro-analytical unit at the National Research Centre, Giza, Egypt.

### 3.2. Methods

#### 3.2.1. Preparation of *N*-Benzoyl-4-(*N*-maleimido)phenyl hydrazide (BMPH)

BMPH was synthesized from maleic anhydride, *p*-aminobenzoic acid and benzhydrazide, as shown in Scheme 1. Maleic anhydride (1 mol) and *p*-aminobenzoic acid (1 mol) were dissolved in DMF 320 mL), then the mixture was stirred at room temperature for 5 h under nitrogen atmosphere. The resulting solution was then poured into a large amount of water to precipitate crude *N*-(4-carboxyphenyl)maleamic acid, which was filtered, dried and recrystallized from water. Yield = 97%; m.p. 223–225 °C (lit. [34] m.p. 225–226 °C).

A mixture of *N*-(4-carboxyphenyl)maleamic acid (0.2 mol), acetic anhydride (100 mL) and fused sodium acetate (2.5 g) was stirred at 55–60 °C for 2 h. The reaction mixture was poured onto a large amount of water to give crude *N*-(4-carboxyphenyl)maleimide, which was filtered and washed with water, dried and recrystallized from methanol: water (6:1) mixture. Yield = 85%; m.p. 211–212 °C (lit. [35] m.p. 208–210 °C). 

A mixture of *N*-(4-carboxyphenyl)maleimide (0.16 mol), thionyl chloride (4.02 mol) and *tert*-butylcatechol (0.01 g) was refluxed for 2 h. Unreacted thionyl chloride was evaporated out, and then the residual product was recrystallized from benzene to obtain pure *N*-[4-(chloro-carbonyl)phenyl]maleimide. Yield = 73.3%; m.p. 166–167 °C (lit. [34] m.p. 168–169 °C).

A solution of ethyl benzoate (0.2 mol) in absolute ethanol (200 mL) was treated with an excess of hydrazine hydrate (20 mL). The reaction mixture was heated under reflux with constant stirring for 3 h, and the resulting solution was cooled in the refrigerator for 24 h. Benzhydrazide was separated as solid particles, filtered and dried under vacuum. It was recrystallized from ethanol. Yield = 94%; m.p. 116–117 °C (lit. [27] m.p. 115–117 °C).

A solution of 5.44 g (0.04 mol) benzhydrazide dissolved in 100 mL DMF was stirred well, and allowed to cool at −10 °C using ice-salt bath for 15 min. Then 9.42 g (0.04 mol) solid *N*-(4-chloro-carbonylphenyl)maleimide was added slowly with constant stirring for 1 h. Ice-salt bath was removed to let the temperature of condensation reaction rise gradually to room temperature and maintained for additional 2 h with stirring. The reaction mixture was slowly poured onto methanol-water mixture (1:2), upon which a white precipitate of BMPH is immediately formed. The product was filtered, dried and recrystallized from 1:1 methanol-water mixture.

#### 3.2.2. Preparation of Stabilized PVC Samples

Samples of PVC for thermal degradation were prepared by thoroughly mixing 1 g of PVC powder with 2 mass % of the stabilizer (or mixed stabilizers) in a mortar and 0.2 g of the resulting fine powder was used in the investigation.

#### 3.2.3. Method of Evaluation of the Stabilizing Efficiency

Evaluation of the stabilizing efficiency was carried out by measuring the rate of dehydrochlorination using a continuous potentiometric determination of the evolved hydrogen chloride gas at 180 °C in air. A detailed description of this method was given elsewhere [36]. The extent of discoloration of the degraded PVC samples was evaluated visually as a function of degradation time. The results obtained are the average of three comparable experiments in each case.

#### 3.2.4. Thermogravimetric Analysis

Thermogravimetric analysis (TG) measurements were made with a Shimadzu TG-50 H thermal analyzer system. Samples were heated from 0 to 500 °C in a platinum pan at a heating rate of 10 °C min^−1^ in a nitrogen atmosphere (30 mL min^−1^).

#### 3.2.5. Molecular Mass Determination by GPC

Average molecular mass of PVC was determined using GPC - HPLC, Waters 600 System controller, 717 plus autosampler. Columns: Phenomenex Phenogel 5 μm 50 A, 300 × 7.8 mm; Detection: Waters model 2410 refractive index; ATTN = 16 × Eluent: THF (100% by Vol.); Flow rate: 0.7 mL min^−1^; Temperature: 50 °C; Injection volume: 25 μL. *Standards*: Polystyrene (PS) 25,000; 13,000; 4000; 2500; 200 g mol^−1^ (1.0% m v ^−1^). Cubic fit calibration curve by Waters Millennium 32 GPC System Software. *Samples*: Dissolved in THF at an approximate 1.0% m v^−1^ concentration.

#### 3.2.6. Antimicrobial Activity of BMPH

Antimicrobial activity of the investigated sample was determined using a modified Kibry-Baur disc diffusion method [37,38,39,40] at Cairo University-Microanalytical Center. The disk diffusion method for filamentous fungi was performed using the approved standard method (M38-A) developed by researchers [41] for evaluating the susceptibilities of filamentous fungi to antifungal agents. The disc diffusion method for yeasts was performed using the standard method (M44-P) approved by the NCCLS [42]. Agar-based methods such as E-test and disk diffusion can be good alternatives because they are simpler and faster than broth-based methods [43,44].

## 4. Conclusions

BMPH is an efficient stabilizer for the thermal degradation of rigid PVC relative to other common industrial reference stabilizers such as DBLC and Ca-Zn stearate. Its greater stabilizing efficiency is based on its longer thermal stability value and its better extent of discoloration and its lower extent of chain scission together with no gel formation which lead to preservation of both the mechanical and physical properties of the polymer. TGA studies indicated that BMPH improved the thermal stability of PVC as judged by the increase in its IDT and by the decrease in its mass losses (%) at a particular temperature. Mixing BMPH with two reference stabilizers in different proportions greatly improves the thermal stability values and also the extent of discoloration. The maximum synergism was obtained in the equivalent weight ratio of BMPH and any of the reference stabilizers. Thus, it is possible to recommend the use of BMPH as a thermal stabilizer for rigid PVC, either alone or as a co-stabilizer with various industrial organometallic stabilizers. Moreover, BMPH stabilizer exhibited reasonable biological activity and it is clear that its antibacterial activity is higher than its antifungal activity. Thus, it can be used as a useful additive for PVC in biomedical and clinical fields.

## Figures and Tables

**Table 1:** Thermal stability values of rigid PVC thermally degraded at 180 °C in air, in the presence of BMPH, reference stabilizers and mixed stabilizers.

Type of stabilizer	Ts/min
Blank PVC	0
DBLC	7
Ca-Zn stearate	6
BMPH	22
BMPH + Ca-Zn stearate (75%:25%)	30
BMPH + Ca-Zn stearate (50%:50%)	50
BMPH + Ca-Zn stearate (25%:75%)	35
BMPH + DBLC (75%:25%)	31
BMPH + DBLC (50%:50%)	50
BMPH + DBLC (25%:75%)	30

**Table 2:** Extent of discoloration of thermally degraded rigid PVC at 180 °C in air for various time intervals, in presence of different stabilizers.

Type of stabilizer	Color at 0 min	Color at 15 min	Color at 25 min	Color at 35 min	Color at 45 min
**Blank PVC**	White	Light brown	Dark brown	Black	Black
**DBLC**	White	Light brown	Brown	Dark brown	Dark brown
**Ca-Zn stearate**	White	Orange	Light brown	Dark brown	Dark brown
**BMPH**	White	yellow	yellow	Dark yellow	Light brown

**Table 3:** GPC measurements of degraded PVC samples.

Sample	Degradation Time/min	M_w_ (g/mol) × 10^4^	M_n_ (g/mol) × 10^4^	PD
**PVC Blank**	0	24.473	9.1520	2.674
**PVC**	30	18.702	4.2958	4.3535
**PVC + BMPH**	30	20.570	6.0777	3.3845

**Table 4:** Thermogravimetric data of blank PVC and PVC in presence of BMPH stabilizer.

**T (°C)**	**Blank PVC**	**PVC + BMPH**
**Initial Decomposition Temperature (*IDT*)**	180 °C	265 °C
**T (°C)**	**Mass loss (%)**	
**200**	5	0
**250**	11	0
**280**	32	23
**300**	44	40
**350**	61	53
**400**	65	60

**Table 5:** Extent of discoloration of rigid PVC thermally degraded at 180 °C in air, for 60 min, in the presence of different weight ratios of stabilizers.

**Mixed stabilizer**	**BMPH + DBLC** **0%:100%**	**BMPH + DBLC** **25%:75%**	**BMPH + DBLC** **50%:50%**	**BMPH + DBLC** **75%:25%**	**BMPH + DBLC** **100%:0%**
Color	Very dark brown	Brown	Yellow	Dark yellow	Dark yellow
**Mixed stabilizer**	**BMPH + Ca-Zn stearate** **0%:100%**	**BMPH + Ca-Zn stearate** **25%:75%**	**BMPH + Ca-Zn stearate** **50%:50%**	**BMPH + Ca-Zn stearate** **75%:25%**	**BMPH + Ca-Zn stearate** **100%:0%**
Color	Dark brown	Brown	Yellow	Dark yellow	Dark yellow

**Table 6:** Antimicrobial activity of BMPH against some bacteria and fungi.

	*S. pneumonia* *G (+ve)*	*S. aureus* *G (+ve)*	*S. typhimurium* *G (-ve)*	*E. coli* *G (-ve)*	*C. albicans*	*A. flavus*
BMPH	26	16	24	20	21	15
Ciprofloxacin (100 µg/mL) (Antibacterial agent)	50	40	40	34		
Flucanazole (100 µg/mL) (Antifungal agent)					45	55
